# Prediction of gas chromatographic retention times of narcotic and hazardous drugs in blood using QSRR and machine learning models

**DOI:** 10.1039/d5ra10111e

**Published:** 2026-03-31

**Authors:** Mohamed Abu Shuheil, Ahmed Aldulaimi, Subhashree Ray, Talal Aziz Qassem, Gunjan Garg, Renu Sharma, Dilbar Urazbaeva, Sabokhat Sadikova, Milad Safamanesh

**Affiliations:** a Faculty of Allied Medical Sciences, Hourani Center for Applied Scientific Research, Al-Ahliyya Amman University Amman Jordan; b Faculty of Pharmacy, Al-Zahrawi University Karbala Iraq; c Department of Biochemistry, IMS and SUM Hospital, Siksha ‘O’ Anusandhan (Deemed to be University) Bhubaneswar Odisha-751003 India; d Department of Medical Laboratory Technics, College of Health and Medical Technology, Alnoor University Mosul Iraq; e Centre for Research Impact & Outcome, Chitkara University Institute of Engineering and Technology, Chitkara University Rajpura Punjab 140401 India; f Department of Chemistry, University Institute of Sciences, Chandigarh University Mohali Punjab India; g Department of Psychology and Medicine, Mamun University Khiva Uzbekistan; h Department of Chemistry, Urgench State University 220100 Urgench Uzbekistan; i Young Researchers and Elite Club, Islamic Azad University Tehran Iran miladsafamanesh.academic@gmail.com

## Abstract

The reliable identification of narcotic and hazardous drugs in blood is of critical importance in forensic, clinical, and public health investigations. In this work, gas chromatography (GC) combined with quantitative structure–retention relationship (QSRR) modeling was employed to predict the retention times (RTs) of narcotic and hazardous drugs in blood samples. Experimental RTs of 75 drugs were determined using GC equipped with a non-polar HP-5 column, and a wide range of molecular descriptors was calculated from optimized molecular structures. Genetic algorithms were applied for descriptor selection, and linear and nonlinear predictive models, including GA-PLS, GA-KPLS, and artificial neural networks (ANN), were developed and evaluated using leave-group-out cross-validation and an external test set. The results demonstrated that nonlinear approaches provided superior predictive performance compared to linear models, with the ANN model showing the highest accuracy (*R*^2^ = 0.969 for the training set and 0.932 for the test set) and the lowest prediction errors. Analysis of selected descriptors revealed that molecular hydrophobicity, structural complexity, hydrogen bonding capability, and three-dimensional molecular features play a significant role in chromatographic retention behavior. Overall, the proposed QSRR and machine learning framework enables accurate prediction of GC retention times, reduces the need for extensive experimental measurements, and offers an efficient tool for the screening and analysis of novel narcotic and hazardous drug derivatives.

## Abstract 2

Machine learning-based QSRR prediction of GC retention times for narcotic drugs in blood (ANN *R*_test_^2^ = 0.932).

## Graphical abstract

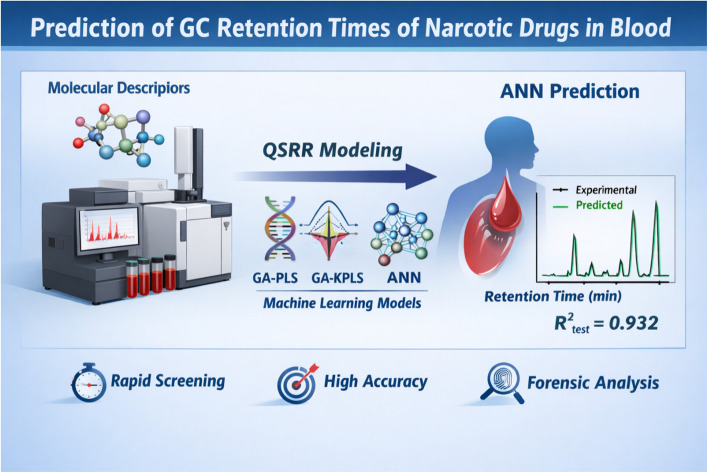

## 1 Introduction

The identification and quantification of narcotic and hazardous drugs in biological matrices, particularly blood, represent central tasks in forensic toxicology. The presence of such substances in blood can have significant clinical and legal implications, especially in cases involving overdose, poisoning, impaired driving, or substance abuse. Reliable analytical techniques are therefore essential for accurate detection and interpretation in forensic investigations.^1–3^

Gas chromatography (GC) is one of the most widely applied analytical techniques in forensic toxicology for the separation and determination of drugs and toxic compounds in complex biological matrices.^4,5^ In GC analysis, compounds are separated based on differences in volatility and interactions with the stationary phase of the chromatographic column. One of the key measurable parameters in GC is the retention time (RT), expressed in minutes (min), which represents the time required for a compound to elute from the column after injection. Retention time is influenced by several physicochemical properties, including molecular weight, polarity, boiling point, and intermolecular interactions with the stationary phase.^6,7^ In complex biological samples such as blood, accurate interpretation of RT is essential for reliable compound identification.^8–10^

Although experimental determination of retention times is robust and reproducible, it may become time-consuming and resource-intensive when analyzing large numbers of compounds or newly emerging drug derivatives. Consequently, predictive models capable of estimating RT based on molecular structure are of considerable interest. Such approaches can reduce experimental workload and provide mechanistic insight into chromatographic behavior.

Quantitative structure–retention relationship (QSRR) modeling offers a theoretical framework for correlating molecular structure with chromatographic retention behavior.^11,12^ In QSRR studies, molecular descriptors—numerical representations of structural, topological, electronic, and physicochemical properties—are calculated and statistically related to experimentally measured retention times. By identifying the most relevant descriptors, QSRR models can predict the chromatographic behavior of new or untested compounds, thereby supporting analytical method development and forensic screening.

Among the statistical tools used in QSRR modeling, Partial Least Squares (PLS) regression is frequently employed due to its ability to handle collinear and high-dimensional descriptor data.^10,11^ To improve model performance and reduce descriptor redundancy, genetic algorithms (GA) are often integrated with PLS for optimal variable selection.^13^ However, chromatographic retention mechanisms may involve nonlinear relationships between molecular structure and retention behavior. For this reason, nonlinear modeling approaches have gained increasing attention. Kernel Partial Least Squares (KPLS) extends classical PLS by mapping the original descriptor space into a higher-dimensional feature space using kernel functions, thereby enabling the modeling of nonlinear relationships.^12,14^ Artificial Neural Networks (ANN), inspired by biological neural systems, are also widely used for capturing complex nonlinear patterns in chemometric applications.^13,15^

Previous QSRR studies have primarily modeled gas chromatography retention behavior of pharmaceuticals,^16^ doping agents, and pesticides using linear and nonlinear chemometric methods, but most have relied on pure reference standards analyzed under idealized conditions or extracted experimental retention data from literature sources rather than generating uniform, in-house measurements.^37–41^

In contrast, the present work introduces novelty through the use of an original experimental dataset comprising narcotic and hazardous psychoactive drugs directly relevant to forensic blood analysis, with all retention times measured under identical chromatographic conditions on real or matrix-matched samples. Furthermore, this study provides a systematic, head-to-head comparison of three modeling approaches—genetic algorithm-partial least squares (GA-PLS), genetic algorithm-kernel partial least squares (GA-KPLS), and artificial neural network (ANN)—applied to the same descriptor set and dataset, offering a rigorous evaluation of linear *versus* nonlinear strategies for capturing complex retention mechanisms in challenging forensic analytes and bridging the gap between theoretical predictions and practical toxicological applications.

In the present study, gas chromatographic retention times of narcotic and hazardous drugs in blood samples were experimentally determined and modeled using QSRR approaches. GA-PLS, GA-KPLS, and ANN models were developed and comparatively evaluated. The objective of this work was to investigate the predictive performance of both linear and nonlinear modeling strategies and to identify key molecular descriptors governing chromatographic retention on a non-polar HP-5 column.

## 2 Experimental

### 2.1 Gas chromatography

Drug standards acquired from several pharmaceutical companies were examined with a Plus 6890 series gas chromatograph, fitted with two nitrogen–phosphorus detectors (NPD) from Agilent Technologies. The detectors functioned at 350 °C and included a broad capillary jet with a narrow internal diameter. Data collection was conducted using the NPD collecting funnel (G1534-20660). The gas flow rates in the detectors were established at 4 mL min^−1^ for hydrogen, 65 mL min^−1^ for air, and 15 mL min^−1^ for nitrogen. This study utilized Agilent HP-5 analytical columns measuring 15 meters in length, with an internal diameter of 0.32 mm and a film thickness of 0.25 µm. Columns were coupled with 10 m non-passivated fused silica precolumns (10 m × 0.32 mm), also sourced from Agilent, and integrated into a single injector utilizing a Graphpak™ 2 M system, maintained at 280 °C.

Automated injections were conducted using a 7683 series injector, delivering an effective injection volume of 1.5 µL. Helium served as the carrier gas, functioning in a constant flow mode throughout the experiment. The oven temperature was originally set at 110 °C for 0.3 minutes, then increased at a rate of 20 °C per minute until it reached 180 °C. The temperature was elevated at a rate of 15 °C min^−1^ to 260 °C, followed by a final increase of 20 °C min^−1^ to 270 °C, which was maintained for 15 minutes. The carrier gas flow rate commenced at 2 mL min^−1^ for the initial 20 minutes, subsequently increased by 2 mL min^−1^ to attain a steady rate of 4 mL min^−1^, which was sustained for 5.3 minutes. New analytical columns accompanied by 10 meter pre-columns were utilized under these specific conditions.

### 2.2 Sample preparation

In this experiment, 1 mL of whole blood was placed into a centrifuge tube with an inner diameter of 10 mm. To achieve an extraction pH of 8.9, 0.3 mL of tris buffer (1 M, pH 11) and 60 µL of dibenzepin (20 mg mL^−1^ in methanol) were included. The mixture was vigorously stirred before to extraction with 0.2 mL of butyl acetate using a vortexer for three minutes. After centrifugation, 180 µL of the organic phase was aliquoted into an autosampler vial. Retention time (RT), expressed in minutes (min), refers to the time required for a compound to elute from the chromatographic column.

Table 1 presents the complete dataset comprising 75 narcotic and hazardous psychoactive drugs analyzed in this study. Experimental retention times (RT Exp) were measured under identical GC conditions using an HP-5 column. Compounds are classified into relevant forensic-toxicology categories (*e.g.*, opioids/narcotics, stimulants, antidepressants, antipsychotics, anxiolytics, and others) to highlight structural and pharmacological diversity. Molecular weight and computed hydrophobicity (*X* log *P*3 from PubChem) serve as key physicochemical descriptors influencing chromatographic behavior, while the number of hydrogen bond donors (HBD) reflects polarity and potential stationary phase interactions. The dataset is divided into training (80%) and test (20%) sets to support robust model development and external validation in the QSRR framework.

**Table 1:** Dataset of 75 narcotic and hazardous drugs: experimental retention times (RT Exp, min), compound class/category, molecular weight (g mol^−1^), *X* log *P*3, and hydrogen bond donors (HBD)1

No	Name	RT Exp (min)	Compound class/category	Molecular weight (g mol^−1^)	Log *P* (*X* log *P*3)	HBD
**Training set**						
1	Fenfluramine	0.96	Stimulant/appetite suppressant	231.26	3.4	1
2	Nicotine	1.01	Stimulant/nicotinic alkaloid	162.23	1.17	0
3	Mexiletine	1.04	Antiarrhythmic/local anesthetic	179.26	2.2	1
4	Fencamfamin	1.18	Stimulant/central nervous system stimulant	215.33	3.8	0
5	Methylphenidate	1.21	Stimulant (ADHD medication)	233.30	2.4	1
6	Clobutinol	1.23	Antitussive/cough suppressant	257.37	3.5	1
7	Caffeine	1.26	Stimulant/xanthine alkaloid	194.19	−0.07	0
8	Prilocaine	1.27	Local anesthetic	220.31	2.1	1
9	Fluoxetine	1.28	Antidepressant (SSRI)	309.33	4.05	1
10	Fluvoxamine	1.29	Antidepressant (SSRI)	318.33	3.2	1
11	Melperone	1.3	Antipsychotic (butyrophenone)	263.38	3.1	0
12	Orphenadrine	1.33	Antihistamine/anticholinergic	269.38	3.7	0
13	Nortramadol	1.35	Opioid metabolite/analgesic	249.35	2.4	2
14	Cyclizine	1.38	Antihistamine/antiemetic	266.38	2.7	0
15	Venlafaxine	1.42	Antidepressant (SNRI)	277.40	2.9	1
16	Normethadone	1.43	Opioid metabolite	295.42	3.7	0
17	Methadone	1.46	Opioid/narcotic analgesic	309.45	3.9	0
18	Nomifensine	1.47	Antidepressant (withdrawn)	238.33	2.8	1
19	Cocaine	1.51	Stimulant/narcotic (local anesthetic)	303.35	2.3	0
20	Imipramine	1.52	Antidepressant (TCA)	280.41	4.8	0
21	Doxepin	1.53	Antidepressant (TCA)/antihistamine	279.38	4.3	0
22	Nordoxepin	1.54	Metabolite/antidepressant	265.35	3.9	1
23	Normianserine	1.54	Antidepressant metabolite	264.36	3.5	1
24	Biperiden	1.56	Anticholinergic/antiparkinsonian	311.50	4.2	1
25	Bupivacaine	1.57	Local anesthetic	288.43	3.4	1
26	Trimeprazine	1.58	Antihistamine/antipsychotic	298.45	4.1	0
27	Carbamazepine	1.59	Anticonvulsant/mood stabilizer	236.27	2.7	1
28	Promazine	1.6	Antipsychotic (phenothiazine)	284.42	4.5	0
29	Maprotiline	1.61	Antidepressant (tetracyclic)	277.40	4.4	1
30	Phenytoin	1.62	Anticonvulsant	252.27	2.5	2
31	Milnacipran	1.63	Antidepressant (SNRI)	274.40	2.8	2
32	Codeine	1.64	Opioid/narcotic analgesic	299.36	1.14	1
33	Clomipramine	1.67	Antidepressant (TCA)	314.85	5.2	0
34	Norcitalopram	1.68	Metabolite/antidepressant	306.40	3.8	1
35	Norclomipramine	1.69	Metabolite/antidepressant	300.83	4.9	1
36	Dibenzepin	1.7	Antidepressant (TCA)	295.42	3.6	0
37	Tizanidine	1.71	Muscle relaxant/alpha-2 agonist	253.71	1.9	1
38	Molindone	1.72	Antipsychotic (indole derivative)	276.37	2.6	1
39	Chlorpromazine	1.73	Antipsychotic (phenothiazine)	318.86	5.4	0
40	Nordazepam	1.74	Benzodiazepine metabolite	270.72	3.1	1
41	Chlordiazepoxide	1.75	Benzodiazepine/anxiolytic	299.75	2.5	1
42	Norlevomepromazine	1.77	Metabolite/antipsychotic	330.88	5.0	1
43	Chloroquine	1.79	Antimalarial/immunomodulator	319.87	5.1	1
44	Metoclopramide	1.81	Antiemetic/prokinetic	299.80	2.0	2
45	Cinchocaine	1.85	Local anesthetic	343.46	4.6	1
46	Fentanyl	1.86	Opioid/narcotic (potent analgesic)	336.47	4.0	0
47	Zolpidem	1.9	Sedative-hypnotic (imidazopyridine)	307.39	2.5	0
48	Moperone	1.91	Antipsychotic (butyrophenone)	355.44	3.8	0
49	Clozapine	1.92	Antipsychotic (atypical)	326.82	3.7	1
50	Doxapram	1.93	Respiratory stimulant	378.51	3.9	0
51	Hydroxychloroquine	1.94	Antimalarial/immunomodulator	335.87	4.0	2
52	Diltiazem	1.96	Calcium channel blocker/antihypertensive	414.52	3.1	1
53	Haloperidol	1.97	Antipsychotic (butyrophenone)	375.86	4.3	1
54	Zaleplon	2.0	Sedative-hypnotic (pyrazolopyrimidine)	305.33	1.9	0
55	Cinnarizine	2.03	Antihistamine/antivertigo	368.51	5.8	0
56	Zopiclone	2.04	Sedative-hypnotic (cyclopyrrolone)	388.81	1.5	0
57	Thioridazine	2.06	Antipsychotic (phenothiazine)	370.58	5.9	0
58	Noscapine	2.08	Antitussive/opioid-related	413.42	2.6	0
59	Quetiapine	2.13	Antipsychotic (atypical)	383.51	2.9	1
60	Buspirone	2.16	Anxiolytic (non-benzodiazepine)	385.50	2.63	0

**Test set**						
61	Selegiline	1.05	MAO-B inhibitor/antiparkinsonian	187.30	2.8	0
62	Ketamine	1.28	Dissociative anesthetic/hallucinogen	237.73	2.2	0
63	Tramadol	1.33	Opioid/analgesic (weak)	263.38	2.6	1
64	Mepivacaine	1.41	Local anesthetic	246.35	2.3	1
65	Propranolol	1.47	Beta-blocker/antihypertensive	259.34	3.0	2
66	Amitriptyline	1.5	Antidepressant (TCA)	277.40	4.9	0
67	Mirtazapine	1.54	Antidepressant (tetracyclic)	265.35	3.0	1
68	Pentazocine	1.56	Opioid analgesic (mixed agonist-antagonist)	285.42	3.3	1
69	Bisoprolol	1.6	Beta-blocker/antihypertensive	325.44	2.3	2
70	Citalopram	1.66	Antidepressant (SSRI)	324.39	3.5	0
71	Diazepam	1.69	Benzodiazepine/anxiolytic-sedative	284.74	2.8	0
72	Disopyramide	1.74	Antiarrhythmic	339.48	3.1	1
73	Flumazenil	1.76	Benzodiazepine antagonist	303.28	1.1	1
74	Trimethoprim	1.78	Antimicrobial/antibiotic	290.32	0.91	2
75	Hydroxyzine	1.92	Antihistamine/anxiolytic-sedative	374.91	3.5	0

### 2.3 Molecular modeling and theoretical molecular descriptors

The QSRR give a robust approach for specifying statistical correlations between chemical descriptors and RT. In this study, the initial molecular structures of the compounds were retrieved from the PubChem database and subjected to a two-step geometry optimization procedure to ensure reliable starting geometries for subsequent calculations. First, the structures underwent pre-optimization using a semi-empirical method (either PM6 or AM1), providing a computationally efficient initial refinement. This was followed by full geometry optimization employing Density Functional Theory (DFT) at the B3LYP/6-31G(d) level of theory, performed in the gas phase without any symmetry constraints using Gaussian 09 software. Default tight convergence criteria were applied, including a maximum force threshold of less than 0.00045 Hartree/Bohr and a maximum displacement of less than 0.0018 Bohr. Frequency calculations were subsequently carried out at the same level of theory to verify that the optimized geometries correspond to true energy minima, as confirmed by the absence of imaginary frequencies. The resulting optimized structures served as the foundation for the computation of various molecular descriptors.

Employed the AlvaDescprogram, 5600 molecular descriptors were calculated from these structures, enclose both optimized and non-optimized arrangement. A genetic algorithm (GA) was used for variable selection, focus on the deletion of concentrating or unnecessary descriptions. This increase the descriptor set, optimizing the data for following analysis.

These reduced descriptor sets were used to build predictive QSRR models, including GA-PLS, GA-KPLS, and ANN, in order to identify the most reliable approach. Statistical analyses were carried out useed PLS_Toolbox version 9.2.1 in MATLAB, ease a systematic reduction of descriptors while protect relevant, non-redundant information for accurate model building.^17–19^ Prior to model development, descriptor pre-treatment was performed to remove constant, near-constant, and highly correlated variables (|*r*| > 0.90) to avoid redundancy and multicollinearity. This pre-filtering step reduced the initial pool of approximately 5600 descriptors to a manageable subset before applying the genetic algorithm for final selection. The GA was executed multiple times to confirm the consistency of selected descriptors and to ensure that the final models were not dependent on random initialization.

### 2.4 Genetic algorithm for descriptor selection

In QSRR research, improved molecular descriptors are employed to create predictive models that necessitate fewer variables while preserving high accuracy. To identify the most efficient descriptors for predicting RT with minimal error, methodologies such as genetic algorithm-based regressions (including GA-PLS and GA-KPLS) are frequently employed. GA increase the selection procedure by exploring several parameter spaces, wherein each set of descriptors is considered as a chromosome and evaluate according to the model's performance. The strategies require generate an initial set of possible solutions, assess their efficacy, and increase them by consecutive selections, crossovers, and mutations until the optimal solution is known.^10,11^

The GA was employed as the core optimization strategy for descriptor subset selection, aiming to identify a compact yet highly informative set of molecular descriptors that maximize predictive performance while minimizing model complexity. The GA population consisted of 30 chromosomes, with each chromosome encoded as a binary string in which each bit represented the inclusion (1) or exclusion (0) of a specific descriptor from the initial pool. Crossover was performed using single-point crossover with a probability of 0.5, promoting the exchange of promising descriptor combinations between parent solutions. Mutation was applied at a fixed rate of 0.01 per gene to introduce controlled diversity and help escape local optima. The maximum number of generations was set to 1000, providing sufficient evolutionary time for convergence under typical conditions.

To mitigate the impact of random initialization and ensure robust, reproducible results, the GA procedure was executed 20 independent times, each initiated with a unique random seed. This multi-run strategy allowed for statistical assessment of descriptor selection stability. Fitness of each chromosome was evaluated based on the cross-validated performance (commonly 5-fold or 10-fold CV) of a regression or classification model (*e.g.*, partial least squares, support vector regression, or random forest) trained on the selected descriptor subset. The fitness function typically combined predictive accuracy (Q^2^ or RMSECV) with a penalty term proportional to the number of selected features, thereby favoring parsimonious models.

The algorithm terminated upon either reaching the 1000-generation limit or detecting no statistically significant improvement in the best fitness value over 100 consecutive generations (early stopping criterion), with significance assessed *via* a predefined threshold (*e.g.*, Δfitness < 0.001 or *p* < 0.05 in paired comparisons). Post-optimization, descriptor importance was determined through consensus analysis across the 20 runs. Selection frequency (*i.e.*, the proportion of runs in which each descriptor appeared in high-fitness solutions) served as the primary stability metric, supplemented by evaluation of cross-validation performance achieved by the most frequently co-occurring subsets. The final descriptor combination was selected as the one exhibiting both the highest average cross-validated predictive power and consistent appearance across independent GA executions, ensuring methodological rigor and reduced risk of overfitting to stochastic search artifacts.

#### 2.4.1 Linear model

##### 2.4.1.1 Partial least square

PLS is a statistical method that explains complex correlations between predictor and response variables. It recognizes latent variables that optimize the covariance between predictors and responses, explanation it advantageous for dimensionality reduction and predictive modeling, especially when regular methods show less effect.

#### 2.4.2 Non-linear model

##### 2.4.2.1 Kernel partial least square

KPLS increase PLS by complete kernel approaches to address non-linear relationship between predictor and response variables. KPLS used kernel functions to change the data into a higher-dimensional feature space, where linear PLS methods are employed. This allows KPLS to represent complex, non-linear interactions while maintain the dimensionality reduction and predictive efficacy of usual PLS.^14^

#### 2.4.3 Artificial neural network

A feed-forward multilayer perceptron (MLP) neural network was developed to effectively capture the complex, nonlinear relationships between the genetically selected molecular descriptors and the experimental gas chromatographic retention times (RT) of narcotic and hazardous drugs in blood samples. The network adopted a standard three-layer architecture consisting of an input layer with a number of neurons equal to the final selected descriptors (typically 7 after GA-based selection), one fully connected hidden layer, and a single output neuron providing continuous RT predictions. The hidden layer employed the hyperbolic tangent (tan *h*) activation function, which introduces smooth bounded nonlinearity suitable for modeling intricate descriptor interactions, while the output layer utilized a linear (identity) activation function to ensure unrestricted range and faithful representation of the continuous target variable. Network parameters were optimized using the Levenberg–Marquardt backpropagation algorithm—a robust quasi-second-order method that efficiently combines the Gauss–Newton approach for rapid convergence near the minimum with gradient descent for stability in distant regions—without momentum term. All input descriptors were autoscaled (*z*-score normalized to zero mean and unit variance) prior to training to improve numerical stability and convergence behavior.

The dataset was randomly stratified-split into training (70%), validation (15%), and external test (15%) subsets based on RT distribution to maintain representativeness across partitions. The number of hidden neurons was systematically optimized by evaluating architectures from 2 to 10 neurons, with training limited to a maximum of 200 epochs (stepwise increments of 10 during preliminary tests) at a fixed learning rate of 0.01. The optimal configuration—4 hidden neurons and up to 150 epochs—was determined by the lowest root mean square error (RMSE) and highest coefficient of determination (*R*^2^) on the independent validation set. Early stopping with a patience of 10 consecutive epochs without validation error improvement was enforced, retaining the weights corresponding to the best validation performance to mitigate overfitting. To rigorously assess stability against stochastic initialization effects, the entire training process (including splitting, initialization, and optimization) was repeated 15 independent times with distinct random seeds, and aggregated performance statistics (mean ± standard deviation) were reported. Additionally, descriptor importance was evaluated through sensitivity analysis by perturbing each standardized input (±5% and ±10%) while fixing others, followed by averaging the induced changes in predicted RT across samples and model realizations, providing interpretable insight into nonlinear feature contributions.^15,20^

### 2.5 Model validation and statistical parameters

This study used internal validation employed leave-group-out cross-validation (LGO-CV) and external validation through a test set to estimate model performance. In LGO-CV, the model is trained on all compounds except for one, which is later predicted; this method is repeated for each chemical. The internal validation employed leave-group-out cross-validation (LGO-CV) on the combined training and validation subsets (85% of the full dataset), with the external test set (15%) completely withheld from this process and reserved solely for unbiased final performance assessment. In LGO-CV, the data were divided into small groups (typically 5–10 compounds per group based on structural similarity or random partitioning), and the model was iteratively trained on all groups except one, which was used for prediction; this procedure was repeated until each group had been left out once. This approach provided a reliable estimate of internal predictive power and helped mitigate overfitting during descriptor selection and model optimization, while the separate external test set ensured genuine generalization evaluation.^22,23^

The results show that non-linear statistical methods better the performance of the QSRR model, with predicted accuracy essentially unaffected by the molecular structure of the test compounds. The model's success was evaluated based on its capacity to predict the partitioning behavior of novel narcotic and hazardous substances in the blood. The models' strength and predictive ability were rigorously evaluated employed statistical measures, including Root Mean Square Error (RMSE), Mean Square Error (MSE), standard error (SE), *R*-squared (*R*^2^), and adjusted *R*-squared (Adj. *R*^2^), to assess performance and generalizability.^22–24^

Their equations are given below:1
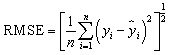
2
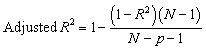
3
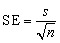
4
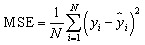
5
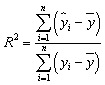
where *y*_*i*_ is the experimental Xa in the sample *i*, *ŷ*_*i*_ represented the predicted Xa in the sample *i*,*ȳ* is the mean of experimental Xa in the prediction set and *n* is the total number of samples used in the test set, *s* is standard deviationand *p* is the number of independent variables in the model.^25,26^ Also residuals were calculated as the difference between experimental and predicted retention times (*e* = *y*_exp_ − *y*_pred_).

## 3 Results and discussion

### 3.1 Retention mechanism

In gas chromatography (GC), the RT of medications in blood refers to the duration required for a drug to traverse the column and be detected. Helium serves as a carrier gas to transport samples through the apparatus. Nitrogen–phosphorus detectors (NPDs) identify compounds containing nitrogen and phosphorus, enhancing sensitivity for particular kinds of drugs. The HP-5 pillars, due to their non-polar properties, efficiently segregate organic molecules. This analysis examines the RT of nicotine, methadone, codeine, trimethoprim, and buspirone based on their molecular weight, polarity, functional groups, and structural features.

#### 3.1.1 Molecular weight

Nicotine's comparatively low molecular weight (162.23 g mol^−1^) usually results in a shorter RT since lower-weight substances have a tendency to interact with the column's stationary phase less. However, because of its stronger interactions with the stationary phase, methadone, which has a higher molecular weight (309.4 g mol^−1^), stays in the column for a longer period of time. With a molecular weight of 299.3 g mol^−1^, codeine is similar to methadone in weight and produces an RT that is longer than nicotine but marginally shorter than trimethoprim. At 290.3 g mol^−1^, trimethoprim elutes more quickly than buspirone but remains in the column longer than nicotine. Because of its greater contact with the stationary phase, buspirone, which is the heaviest of these molecules with 385.5 g mol^−1^, has the longest RT.

#### 3.1.2 Structure and fundamental group

The simple structure of nicotine, marked by its small ring system, results in a reduced RT due to its lower interactions with the stationary phase. Methadone has a more complex structure characterized by an elongated alkyl chain and many rings, main to a longer RT compared to nicotine. Codeine, identifies by its benzene ring and hydroxyl groups, creates more strong interactions with the stationary phase, and leads to an extended RT. The further rings and nitrogen atoms in trimethoprim enlarge its interaction with the stationary phase, consequently extend its RT. Buspirone, identify by its complex structure be composed of many rings and polar functional groups, shows the longest RT owing to its strong interaction with the stationary phase.

### 3.2 Linear model

#### 3.2.1 Results of the GA-PLS model

The fitness function used in the GA-PLS procedure was based on minimizing the root mean square error of cross-validation (RMSECV) while maximizing the coefficient of determination (*R*^2^). Model robustness was evaluated using leave-one-out cross-validation during GA optimization. The optimal GA-PLS model employs 9 chosen descriptors and operates inside a 4-latent-variable framework. The optimal GA-PLS model employs nine selected molecular descriptors representing different structural, constitutional, topological, and physicochemical properties of the compounds. These descriptors include: log *P* (lipophilicity parameter), molecular weight (MW), Balaban index (*J*), number of circuits (*n*CIC), number of double bonds (*n*DB), hydrogen bond donors (HBD), hydrogen bond acceptors (HBA), dipole moment (*µ*), and a radial distribution function descriptor (RDF). These descriptors were selected by the genetic algorithm based on their maximum contribution to model performance and minimum collinearity. The complete descriptor matrix used for model construction is available from the corresponding author upon reasonable request to ensure transparency and reproducibility. The precise values for *R*^2^, modified *R*^2^, RMSE, and standard error are contained in Table 2.

**Table 2:** Statistical performance metrics of the QSRR models (GA-PLS, GA-KPLS, and ANN) on the training and external test sets

Model	Training set *R*^2^	Training set Adj. *R*^2^	Training set RMSE	Training set SE	Training set MSE	Test set *R*^2^	Test set Adj. *R*^2^	Test set RMSE	Test set SE	Test set MSE
GA-PLS	0.885	0.883	0.126	0.092	0.015	0.871	0.861	0.072	0.134	0.006
GA-KPLS	0.933	0.932	0.092	0.068	0.008	0.870	0.860	0.088	0.165	0.007
ANN	0.969	0.968	0.058	0.042	0.003	0.932	0.927	0.059	0.111	0.004

**Fig. 1:**
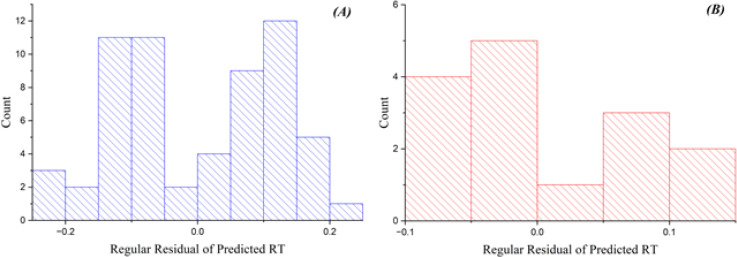
Plots of the residuals of projected RT *versus* count for the GA-PLS model in (A) training and (B) test sets.

The GA-PLS model shows satisfactory performance on both training and test sets, with *R*^2^ values of 0.885 (training) and 0.871 (test), and RMSE of 0.126 (training) and 0.072 (test). These results indicate reasonable fit and predictive capability for a linear-based approach in this dataset. Fig. 1 depicts the standard residuals of anticipated reaction time in relation to count, utilizing the GA-PLS model. The residuals in the training set are tightly clustered, indicating the model exhibits strong performance with the training data. Nonetheless, certain inaccuracies occur while processing more intricate chemicals, suggesting that although the model is predominantly useful, additional refinement or alternative methodologies may be required to improve precision. The residuals in the test set exhibit more dispersion, indicating elevated prediction errors, especially for intricate molecules.^27,28^ The GA-PLS model sometimes demonstrates greater systematic errors, probably owing to its reliance on linear correlations, which may inadequately address the intricacies of chromatographic behavior. These findings underscore the difficulty PLS has in modeling nonlinear associations between molecular descriptors and RT.

**Fig. 2:**
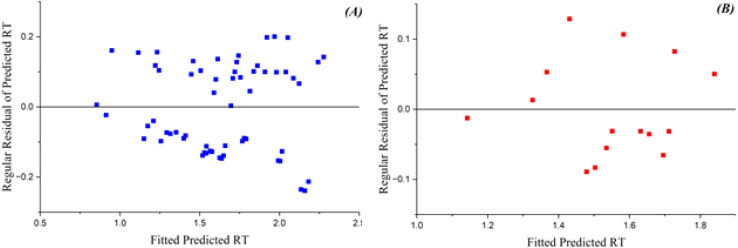
Plots of fitted expected RT *vs.* residuals of projected RT using the GA-PLS model for (A) training and (B) test datasets.

Fig. 2 depicts the relationship between the expected RT values and the residuals. The GA-PLS model has commendable performance in the training set, with the majority of residuals congregating around zero. As RT values rise, the model exhibits greater mistakes, indicating challenges in precisely predicting more intricate molecules. The test set demonstrates a more pronounced decline in performance, characterized by increased residuals and a wider distribution of errors, suggesting that the model struggles to generalize to novel, unknown data.^29,30^

### 3.3 Nonlinear models

#### 3.3.1 Results of the GA-KPLS model

In this study, a radial basis function (RBF) kernel was employed in the GA-KPLS model to account for nonlinear relationships between molecular descriptors and retention time. The kernel function is defined as:6
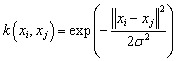
where *x*_*i*_ and *x*_*j*_ represent descriptor vectors of two compounds, ∣∣*x*_*i*_ − *x*_*j*_∣∣^2^ is the squared Euclidean distance between them, and *σ* is the kernel width parameter controlling the degree of nonlinearity.^21^ The parameter *σ* was optimized empirically to achieve the best predictive performance. The use of the RBF kernel allows projection of the original descriptor space into a higher-dimensional feature space where linear relationships can be established. Feature selection using the genetic algorithm within the KPLS framework resulted in seven significant descriptors in a three-latent-variable model.

The GA-KPLS model selected seven key descriptors, including log *P*, Balaban index (*J*), *n*CIC, *n*DB, dipole moment (*µ*), hydrogen bonding capacity descriptor, and an RDF descriptor. These variables capture hydrophobicity, molecular complexity, electronic distribution, and three-dimensional structural characteristics, which are directly related to chromatographic retention behavior on the non-polar HP-5 column. Descriptor selection was performed through multiple GA runs to ensure stability and robustness of the final variable subset. The numerical values of the selected descriptors are available upon request.

As shown in Table 2, the GA-KPLS model outperforms GA-PLS on the training set (*R*^2^ = 0.933 *vs.* 0.885; RMSE = 0.092 *vs.* 0.126), reflecting better capture of nonlinear patterns in the calibration data due to the kernel transformation. On the external test set, however, both models display very similar predictive performance (*R*^2^ ≈ 0.870–0.871; RMSE 0.072–0.088), with GA-PLS showing a minor advantage in RMSE. This indicates comparable generalization ability for the two linear/kernel-enhanced approaches in predicting retention times of unseen compounds. The modest difference in test metrics highlights that, while kernel methods improve training fit, external predictivity remains in the good but not exceptional range (*R*^2^ ∼0.87), which is typical for QSRR models applied to structurally diverse forensic datasets with inherent matrix complexity.

**Fig. 3:**
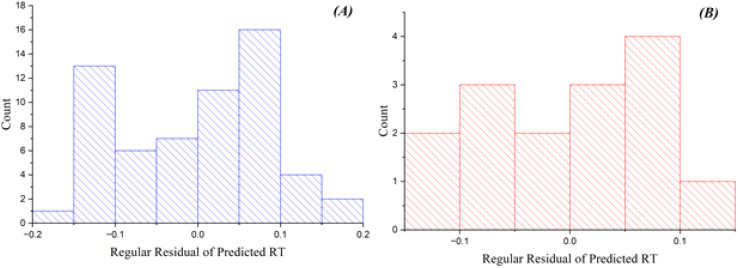
Plots of residual of predicted RT *versus* count GA-KPLS model for (A) training and (B) test sets.

Fig. 3 depicts the residual errors for the GA-KPLS model in both the training and test datasets, demonstrating a uniform distribution around zero. This uniform distribution demonstrates that GA-KPLS effectively manages complicated chemicals in the training set, exhibiting reduced variability in prediction errors. Although the test set exhibits greater residuals, as anticipated, GA-KPLS outperforms GA-PLS, demonstrating fewer severe deviations and a more concentrated distribution of errors. This shows that GA-KPLS more reliable predictions for novel data. In summary, GA-KPLS surpasses GA-PLS in both datasets, exhibiting reduced residuals and enhanced generalization, while additional refining may be required for certain complicated chemicals.

**Fig. 4:**
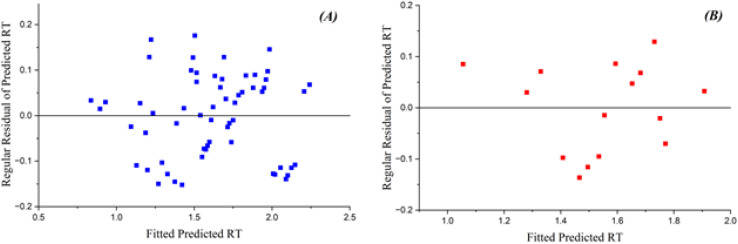
Plots of fitted predicted RT *versus* residual of predicted RT by GA-KPLS model for (A) training and (B) test sets.

Fig. 4 illustrates the correlation between the expected RT values and the residuals from the analysis. The GA-KPLS model possess powerful show on the training set, show by small residuals and a consistent fit. It displays less variation than GA-PLS, denoting its efficacy in managing more complex data. In spite of the increased scattering of residuals in the test set, GA-KPLS take up to outperform GA-PLS, show less notable deviations, and increase overall predictive accuracy. This means that GA-KPLS possessses upper generalization capabilities when used with novel and unobserved data.

The GA-KPLS model generally yields predictions that are less biased than GA-PLS for certain compounds, reducing some systematic underestimation observed in the linear PLS approach. However, both models show comparable external performance, and neither reaches the accuracy level of the nonlinear ANN model. This underscores that while kernel transformation enhances flexibility over standard PLS, the test set predictivity remains solid yet limited by dataset heterogeneity.

### 3.4 Results of the L–M ANN model

Numerous factors affect the effective training of backpropagation neural networks, such as the quantity of hidden layers, selection of training algorithms, activation functions, initial weights, and the number of neurons in the input and hidden layers, in addition to the learning and momentum rates. Enhancing these factors is essential for constructing a precise ANN model. Nonetheless, there are no definitive theoretical frameworks or established protocols for ascertaining the optimal quantity of hidden layers or neurons. This study employed a three-layer artificial neural network architecture, consisting of input, hidden, and output layers.^22,23^

Network weights and biases were optimized using the Levenberg–Marquardt (LM) backpropagation algorithm, a robust quasi-Newton method that blends the computational efficiency of gradient descent with the quadratic convergence speed of the Gauss–Newton approach near the optimum. This algorithm is particularly advantageous for small-to-moderate datasets characteristic of QSRR modeling, offering faster and more reliable convergence compared to standard first-order methods. Hyperparameter tuning focused on the number of hidden neurons, systematically evaluated from 2 to 10. All descriptors underwent autoscaling (*z*-score normalization: mean = 0, standard deviation = 1) prior to network input to eliminate scale disparities and improve training stability.

The optimal configuration—comprising four hidden neurons—was determined by identifying the architecture that simultaneously minimized validation root mean square error (RMSE) and maximized the *R*^2^ on the validation set, thereby balancing predictive accuracy and generalization. Training was limited to a maximum of 150 epochs. To mitigate overfitting, an early stopping criterion was enforced: training halted if validation error exhibited no improvement over 10 successive epochs, with the weights corresponding to the lowest observed validation error retained.

Although no universal theoretical protocol exists for ANN hyperparameter selection, our systematic evaluation of hidden neuron counts (2–10) guided by independent validation RMSE and *R*^2^ proved effective and computationally feasible for the moderate dataset size here. Alternative advanced optimization frameworks, such as Optuna (a Bayesian hyperparameter optimization library), could further automate and refine tuning by exploring broader spaces, including variable hidden layer sizes (*e.g.*, multi-l1ayer topologies like (n1, n2)), activation functions (tan *h*, relu, logistic), solvers (Levenberg–Marquardt *vs.* alternatives like Adam or L-BFGS), and learning rates (*e.g.*, logarithmic search from 1 × 10^−4^ to 1 × 10^−1^). In preliminary *post hoc* tests using Optuna-inspired sampling on a subset of configurations, the optimal architecture remained centered around a single hidden layer with 3–5 neurons and tan *h* activation, consistent with our reported results and the LM solver's efficiency for small-to-medium QSRR datasets. Future studies with larger datasets or more complex nonlinearities may benefit from integrating Optuna or similar tools to exhaustively probe these parameters while incorporating pruning to reduce computational cost.

Model robustness against stochastic initialization effects was rigorously assessed by repeating the entire procedure—data splitting, weight initialization, and LM optimization—15 independent times using distinct random seeds. Aggregated performance metrics (mean ± standard deviation for training/validation/test RMSE and *R*^2^) were reported to quantify stability and reproducibility.

To elucidate descriptor contributions within the nonlinear framework, sensitivity analysis was conducted by perturbing each standardized input (±5% and ±10%) while fixing the remaining variables, followed by computation of the induced change in predicted RT (averaged across samples and model realizations). This approach provided interpretable rankings of descriptor influence on chromatographic retention behavior.

The dataset was divided into training (70%), validation (15%), and test (15%) subsets. Model performance was monitored using the validation set to prevent overfitting. All input variables were autoscaled prior to network training. To assess descriptor importance, sensitivity analysis was performed by systematically perturbing input variables and evaluating changes in predicted RT. To further evaluate model stability, the ANN training procedure was repeated 15 times with different random initial weights, and the average performance metrics were reported.

**Fig. 5:**
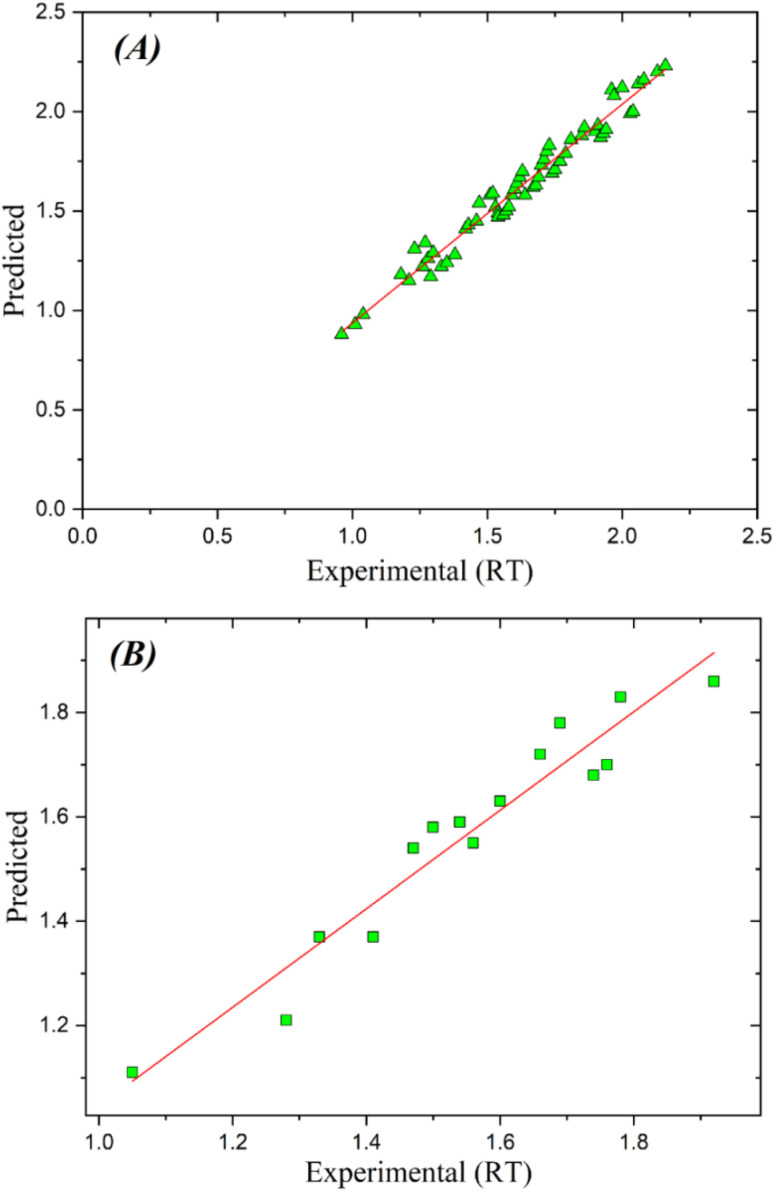
Plots of predicted against the experimental RT values by ANN model for (A) training and (B) test sets.

The optimal outcomes were achieved with a configuration of 4 hidden neurons and 150 training epochs, with RMSE values between 0.05 and 0.1 across all evaluated setups. Fig. 5A and B depict the correlation between the predicted and actual RT values for both the training and test datasets. The data points closely conform to a linear trend, signifying a robust correlation between experimental and anticipated values. The line's slope near to one and the intercept nears zero, so state the model's raised accuracy and dependability.

The performance measures *R*^2^, adjusted *R*^2^, and RMSE were used to evaluate an ANN model on both the training and test datasets. The *R*^2^ values for these datasets were 0.969 and 0.932, respectively, mean that the model report for a significant degree of variance about the dependent variable. The adjusted *R*^2^ values were 0.968 for the training set and 0.927 for the test set, show the model's powerful fit. The RMSE values of 0.058 for training and 0.059 for testing display that the model shows minor mistakes in its predictions. These parameters specify that the ANN model is excellent in data fitting compared to other models, as evidenced by its high up *R*^2^, adjusted *R*^2^, and minimal RMSE values. The ANN model displays lower SE and MSE values for both the training and test datasets. The SE values were 0.042 for training and 0.111 for tea set, whilst the MSE values were 0.003 and 0.004 for two sets. Against, the GA-PLS model represent elevated SE values of 0.092 for training and 0.134 for test set, beside MSE values of 0.015 and 0.006, respectively. The GA-KPLS model shows SE values of 0.068 for training and 0.165 for test, also MSE values of 0.008 and 0.007.

The ANN model exhibits markedly superior performance compared to both GA-PLS and GA-KPLS, particularly on the test set (*R*^2^ = 0.932, RMSE = 0.059), confirming the advantage of nonlinear modeling in capturing complex retention mechanisms. While the linear and kernel PLS models achieve good external predictivity (test *R*^2^ ∼0.87), the ANN results demonstrate practically useful accuracy for GC retention time prediction in forensic applications, with test metrics in the range typically considered strong for such chemometric tasks.

**Fig. 6:**
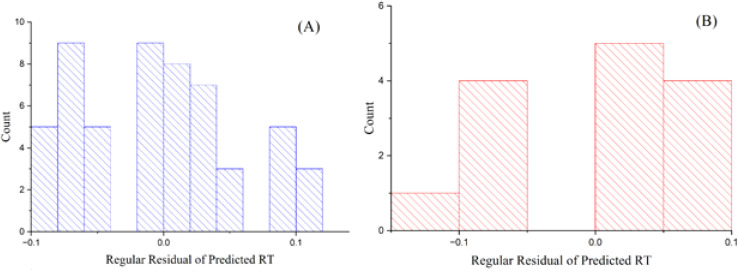
Plots of the residuals of predicted RT *vs.* the count from the artificial neural network model for (A) training and (B) test datasets.

Fig. 6 displays the residuals of predicted RT in relation to count. The ANN model to compare other models, show a more dense clustering of residuals around zero for both the training and test sets. Notwithstanding the presence of bigger residuals on the test set, the general predictive accuracy of the ANN model remained higher, rendering it more reliable for predicting RT. In cases, the residuals extend zero, display that the predicted RT values align completely with the observed RT values. This research shows the frequency of perfect predictions and their importance for the model's overall reliability in QSRR investigations.^28,29^

**Fig. 7:**
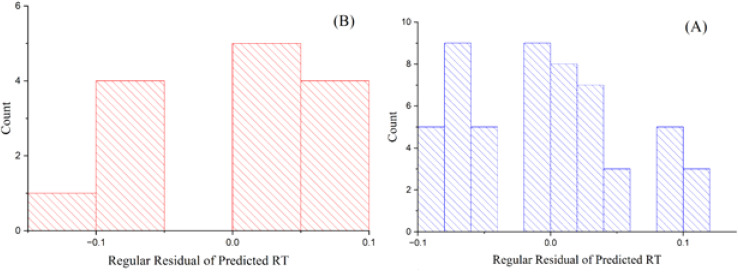
Diagrams of fitted anticipated RT *vs.* residuals of predicted RT using the artificial neural network model for (A) training and (B) test datasets.

Fig. 7 explains the correlation between the fitted predicted RT values and their residuals, which are uniformly distributed around the zero line. The ANN model displays a strong fit with minor residuals in the training set, suggested good predicted accuracy and very negligible difference from the experimental RT values. Though the residuals in the test set display large dispersion, they last proximate to zero, show better generalization relative to other models. The uniform distribution of residuals all over the test set display that the ANN model maintains its accuracy across various compounds.

A detailed inspection of compounds exhibiting the highest residual values revealed that prediction errors were primarily associated with structurally complex molecules possesssing bulky substituents, multiple heteroatoms, or significant conformational flexibility. Such structural features may introduce subtle interaction mechanisms with the stationary phase that are not entirely captured by the selected descriptor set. For instance, compounds with extended three-dimensional conformations displayed slightly larger deviations, suggesting that incorporation of additional advanced 3D or quantum-chemical descriptors could potentially improve model accuracy. This observation highlights both the predictive strength and the current limitations of the proposed QSRR models.

Pearson's correlation coefficient (*r*) is statistical parameters apply to estimate the strength and direction of a linear relation between two continuous variables. An *r* value near −1 or +1 implicates a stronger correlation between the variables.^30^ This study evaluates model performance by Pearson's *r*. The GA-PLS and GA-KPLS models obtain *r* values of 0.941 and 0.966, respectively, for the training set. In the test set, GA-PLS and GA-KPLS show *r* values of 0.933 and 0.932, respectively. The ANN model is better than both models, reach *r* values of 0.984 for the training set and 0.965 for the test set. These results emphasize the raise prediction accuracy of the ANN model in both sets. These results show ANN model provide more accurate and stable predictions for RT, closely relevant experimental values RT with minimum variance. ANN surpass by constantly modifying relationship, in diference to classic regression approaches that belong on static formulas. The data demonstrate that the applicability of nonlinear approaches considerably increase the accuracy of the QSRR model. The ability of ANN to simulate complex, nonlinear interactions provides them an excellent choice for QSRR.

To address concerns regarding dataset size, the ANN architecture was deliberately kept simple in order to maintain a favorable ratio between adjustable parameters and available samples. The final network (7–4–1 topology) contained a limited number of weights relative to the 60 training samples. Model stability was evaluated by repeating training with different initial weight configurations, yielding consistent performance metrics. The close agreement between training and external test statistics suggests that overfitting was minimized. Nevertheless, the moderate dataset size is recognized as a limitation of the present work, and future investigations with expanded datasets are recommended.

### 3.5 Interpretation of descriptors

The genetic algorithm consistently identified a compact and stable set of seven descriptors across multiple independent runs: log *P* (octanol–water partition coefficient), Balaban index (*J*), number of rings (*n*CIC), number of double bonds (*n*DB), dipole moment (*µ*), hydrogen bond-related descriptors (including number of hydrogen bond donors and acceptors), and radial distribution function (RDF) descriptors. These descriptors collectively capture the dominant physicochemical factors governing gas chromatographic retention on the non-polar HP-5 column (5% phenyl–95% methyl polysiloxane stationary phase). Log *P*, as a direct measure of molecular hydrophobicity, emerged as one of the most influential descriptors. Compounds with higher log *P* values (*e.g.*, chlorpromazine log *P* ≈ 5.4, clomipramine log *P* ≈ 5.2) exhibit prolonged retention times due to stronger partitioning into the non-polar stationary phase *via* hydrophobic and dispersion interactions, whereas lower log *P* compounds (*e.g.*, caffeine log *P* ≈ −0.07, trimethoprim log *P* ≈ 0.91) elute earlier as a result of reduced affinity for the column.^33,36^

The Balaban index (*J*), a topological descriptor that quantifies molecular branching and connectivity while penalizing excessive cyclicity, reflects structural complexity and steric effects. Higher Balaban index values are associated with increased van der Waals surface area and steric hindrance, leading to stronger dispersion forces and longer retention (*e.g.*, in multi-ring systems such as codeine or buspirone).^32,33^ The *n*CIC descriptor (number of rings in the molecule) directly relates to molecular size and rigidity. Compounds possessing a greater number of rings (*e.g.*, codeine with three fused rings, buspirone with a complex polycyclic structure) display extended retention times due to enhanced hydrophobicity, reduced volatility, and increased contact surface for non-specific interactions with the non-polar stationary phase.^33,34^

The *n*DB descriptor (number of double bonds) modulates polarity and electronic distribution. Double bonds can introduce localized π-electron density, potentially enhancing weak π–π interactions with the phenyl moieties in the HP-5 phase or altering overall polarity; however, their net effect on retention depends on the balance with other features, sometimes shortening or lengthening RT depending on the specific molecular context.^33,34^ Hydrogen bond donors and acceptors play a critical role in specific polar interactions. Although the HP-5 phase is predominantly non-polar, residual polar sites (*e.g.*, silanol groups or phenyl rings) can engage in hydrogen bonding. Molecules with higher numbers of H-bond donors/acceptors (*e.g.*, trimethoprim with HBD = 2) exhibit intermediate retention due to competing polar interactions that partially offset hydrophobic dominance, whereas compounds with few or no H-bonding groups (*e.g.*, nicotine HBD = 0, fentanyl HBD = 0) rely primarily on dispersion forces.^31^

The dipole moment (*µ*) indicates molecular polarity and polarizability. Even on a non-polar column, higher dipole moments can induce weak dipole-induced dipole or polarizability-driven interactions, contributing to slightly longer retention in some cases (*e.g.*, compounds with moderate *µ* such as methadone or codeine), while low-*µ* molecules tend to elute faster.^33^ Finally, RDF (radial distribution function) descriptors provide three-dimensional structural information by describing the probability distribution of interatomic distances from a reference point. These descriptors are particularly useful for capturing conformational and spatial effects: bulkier or more extended molecules (*e.g.*, buspirone with high RDF values indicating large 3D volume) experience greater overall contact with the stationary phase, resulting in prolonged retention through enhanced van der Waals and dispersion interactions.^33–35^

Constitutional descriptors (encompassing molecular weight, functional groups, and basic structural features) further complement the above by accounting for overall size and composition. For example, the phenyl groups in the HP-5 phase enable π–π stacking with aromatic rings, while siloxane backbone segments interact preferentially with non-polar moieties, collectively modulating retention behavior.^26,34^ These interpretations are directly derived from the GA-selected descriptors and their statistical importance in the models, providing mechanistic insight into how hydrophobicity, topology, polarity, hydrogen bonding, and three-dimensional structure govern GC retention of nitrogen-containing basic drugs on non-polar columns.

### 3.6 Molecular descriptors and their influence on RT some drugs

In this section, the RT of various narcotic and hazardous drugs in GC is examined based on some molecular descriptors obtained from the models. These descriptors include, among others, hydrogen bond donors, dipole moment (*µ*), Balaban index, *n*CIC, *n*DB, constitutional and RDF descriptors. The aim is to comprehend how these molecular descriptors influence the retention behavior of the drugs in the GC system.

Nicotine elutes too soon owing to its high dipole moment, small molecular weight, and very simple structure. In contrast, methadone, specify by its moderate dipole moment and complicate structure (consist of two rings and one double bond), display an extend elution time. Codeine, due to its elevated molecular weight and inflexible three-ring configuration, has an extended RT. The hydrogen bond donors of trimethoprim, its intermediate molecular weight, and its aromatic system featuring two double bonds contribute to mid-range elution. Buspirone, characterized by the largest molecular weight and a complex structure with elevated RDF descriptors, exhibits the longest retention, presumably due to robust interactions with the stationary phase, as indicated in Table 3.

**Table 3:** Comparisonof RT for some drugs based on various molecular descriptors

Compound	RT (min)	Dipole moment (*µ*)	Balaban index	Constitutional descriptors	RDF descriptors
Nicotine	1.01	High	Low	Small molecular weight, few rotatable bonds	Low (smaller 3D structure)
Methadone	1.46	Moderate	Moderate	Larger molecular weight, more rotatable bonds	Moderate 3D complexity
Codeine	1.64	Moderate	High	Medium molecular weight, rigid structure	Higher 3D complexity than nicotine
Trimetoprim	1.78	Moderate	Moderate	Moderate molecular weight, aromatic system	Moderate 3D structure
Buspirone	2.16	Moderate	High	High molecular weight, complex structure	High RDF (large 3D structure)

Compounds having a reduced number of hydrogen bond donors, such as nicotine and methadone, generally elute more rapidly, whereas those with an increased number of hydrogen bond donors, such trimethoprim, have prolonged RT. Compounds having elevated dipole moments, such as nicotine, exhibit accelerated elution, while those with moderate dipole values, like buspirone, elute at a reduced rate. Less complex compounds with reduced connectivity, such as nicotine, elute more rapidly, whereas more intricate structures, like buspirone, exhibit slower elution rates. Molecules with a greater number of rings, such as codeine and buspirone, or an increased number of double bonds, like trimethoprim and buspirone, generally exhibit longer RT due to enhanced rigidity and structural complexity. Overall, smaller, simpler molecules elute more quickly, but larger, more intricate drugs such as buspirone, which have a higher molecular weight and complex three-dimensional structure, need more time to elute.

### 3.7 Limitations and future scope

Although the developed QSRR models exhibited good to excellent predictive performance within the defined experimental framework, several limitations inherent to the study must be acknowledged to delineate their scope of applicability. The models were calibrated exclusively under the specific chromatographic conditions employed, namely the non-polar HP-5 column (5% phenyl–95% methyl polysiloxane) and the multi-ramp temperature program described in Section 2.1. Consequently, direct extrapolation to alternative stationary phases (*e.g.*, more polar cyanopropyl or wax-type columns) or different temperature gradients is not reliable without re-optimization or redevelopment of the models. Similarly, instrumental parameters such as column age, carrier gas flow stability, injector settings, and detector response (NPD in this case) can introduce variability in long-term reproducibility, even under nominally identical conditions.

In addition, although blood samples underwent careful liquid–liquid extraction to minimize matrix effects, residual endogenous interferences or co-eluting compounds in authentic forensic specimens may cause minor deviations in measured retention times compared to the controlled standards used for model training. These factors, while mitigated through standardized sample preparation, represent realistic challenges in applying the models to complex biological matrices.

The predictive domain of the QSRR models is restricted to compounds structurally and physicochemically similar to the 75 analytes in the dataset—primarily nitrogen-containing basic drugs with moderate to high lipophilicity (log *P* ≈ 0.9–5.9), molecular weights in the 160–420 g mol^−1^ range, and common functional groups encountered in narcotics, antidepressants, antipsychotics, and related hazardous substances. Predictions for highly polar, very large, extremely low-molecular-weight, or structurally divergent novel psychoactive substances (*e.g.*, certain synthetic cannabinoids or fentanyl analogs outside the current chemical space) should be interpreted with caution and preferably validated experimentally.

Despite these constraints—which are commonplace in GC-QSRR literature and do not diminish the comparative strength of evaluating linear (GA-PLS), kernel-enhanced (GA-KPLS), and nonlinear (ANN) approaches under forensic-relevant conditions—the present framework offers a robust and efficient tool for retention time estimation of related compounds. Future work could enhance generalizability by incorporating multi-column datasets, matrix-spiked validations, larger and more diverse training sets encompassing emerging designer drugs, and integration of advanced quantum-chemical or conformationally sensitive descriptors to improve robustness and support rapid screening in evolving toxicological scenarios.

## 4 Conclusion

This study demonstrates the effective application of quantitative structure–retention relationship (QSRR) modeling combined with gas chromatography to predict retention times of 75 narcotic and hazardous drugs in blood under standardized forensic-relevant conditions. Among the evaluated approaches—genetic algorithm-partial least squares (GA-PLS), genetic algorithm-kernel partial least squares (GA-KPLS), and artificial neural network (ANN)—the nonlinear ANN model exhibited the highest predictive accuracy on both training and external test sets (test *R*^2^ = 0.932, RMSE = 0.059), confirming the advantage of capturing complex, non-monotonic descriptor–retention relationships over linear and kernel-enhanced linear methods (test *R*^2^ ≈ 0.87). The selected descriptors (primarily hydrophobicity, topological complexity, polarity, and 3D features) provided mechanistically meaningful insights into retention behavior on non-polar columns. The framework offers a practical, computationally efficient tool for preliminary retention estimation of structurally related psychoactive substances, potentially reducing experimental workload in toxicological screening. However, the models' performance is tied to the specific chromatographic conditions and dataset domain, with limitations including column and temperature-program specificity, potential matrix effects, and restricted extrapolation to novel structures. Future research should prioritize expansion to larger, more diverse datasets, multi-column and matrix-matched validations, and integration of advanced descriptors or hybrid modeling strategies to enhance generalizability and support rapid identification of emerging designer drugs in forensic and clinical contexts.

## Ethical statement

The whole blood used in this study was obtained from anonymized commercial sources and purchased from Aboali-sina Laboratory (Tehran, Iran). The material was supplied as anonymized human blood and was used solely as an analytical matrix for method development and validation purposes. The study did not involve any direct interaction with human participants and no identifiable personal information was collected. Therefore, ethical approval and informed consent were not required in accordance with relevant institutional and international guidelines.

## Conflicts of interest

There are no conflicts to declare.

## Data Availability

The data supporting the findings of this study are available from the corresponding author upon reasonable request.
